# Patient Preferences with Regard to Laser versus Intravitreal Injections in the Treatment of Diabetic Macular Edema

**DOI:** 10.1155/2017/7398470

**Published:** 2017-04-04

**Authors:** Lauren Mason, Jason N. Crosson, John O. Mason, Gerald McGwin

**Affiliations:** Retina Consultants of Alabama, University of Alabama at Birmingham School of Medicine, Callahan Eye Hospital, 700 18th St. South, Suite 707, Birmingham, AL 35233, USA

## Abstract

*Purpose*. To identify treatment preferences of patients with diabetic macular edema (DME) having undergone laser and intravitreal injections. *Methods*. Patients with DME who received lasers and injections were surveyed, measuring preferences toward specific treatments. 66/210 diabetic patients met the criteria for our survey assessing preference for lasers and/or injections, incorporating demographics and treatment preference questions. Outcome measures included treatment preference (laser or injections), how often patients are willing to be treated, and how much vision they will sacrifice to avoid being treated every month. *Results*. 66 patients completed the survey. The mean diabetes duration was 20.7 years, the mean retina follow-up was 4.4 years, and patients received a mean of 4.82 lasers and 4.86 injections. 56% preferred injections, 33% preferred laser, and 11% had no preference. Regarding treatment effectiveness, 38% found no difference, 36% chose laser, and 25% chose injections. Regarding anxiety, 56% reported injection anxiety. While 50% versus 38% reported that laser was easier than injections. 91% would give up zero lines on the eye chart, and 76% would come in 12 times yearly for treatment to maintain vision. *Conclusion*. Patients with DME have no profound preference regarding laser versus intravitreal injections but prefer aggressive treatment and are unwilling to sacrifice vision for less visits.

## 1. Introduction

Diabetic mellitus is quickly becoming an epidemic in the United States, with an estimated 29.1 million Americans diagnosed to date [1]. Visual complications that result from diabetes, such as diabetic retinopathy and diabetic macular edema, remain the primary causes of vision loss in the U.S. Diabetic macular edema (DME) is a common progressive eye disease that affects an estimated 750,000 American diabetic patients [2]. Because of this, DME has an enormous impact on the quality of life in these patients, affecting their ability to perform everyday tasks such as driving and holding a job, with resulting depression and anxiety [3]. Due to the growing number of diabetic patients being diagnosed with DME, studies have been done to examine current treatment protocols for these patients to determine the most effective way to maintain visual stability and improvement over a long-term treatment plan. Although there are studies such as Miller et al. that have looked at treatment recommendations for DME based on physician preference [4], no study to date has evaluated actual patient preferences with regard to focal grid laser and intravitreal injections. The objective of our study was to perform a patient survey to identify the treatment preferences of patients with DME that had already been treated with both focal grid laser and bevacizumab intravitreal injections. Furthermore, we wished to identify how often patients are willing to be treated, which treatment they prefer (laser or injections), and how much vision they are willing to lose to avoid being treated every month.

## 2. Methods

After IRB approval, we conducted a single-center patient preference survey on patients with DME who had previously been treated with both focal grid lasers and bevacizumab intravitreal injections.

We consecutively evaluated 210 patients with diabetic retinopathy and found 66 who met entry criteria for our survey study. The entry criteria were patients over the age of 18 who could give informed consent to participate and who had prior focal grid laser treatment and intravitreal injections for DME. Exclusion criteria included prior panretinal photocoagulation (PRP) for proliferative diabetic retinopathy (PDR). Patients were given a 21-question survey (Table 1) that assessed patient preference for focal grid lasers and/or intravitreal injections. The survey included demographic features such as age, race, gender, socioeconomic status, transportation issues, employment and education, insurance provider, and disability status to analyze whether these factors correlated with the results regarding the primary outcome measures as listed on the survey.

Survey questions included the following: which treatment, focal grid laser or injection, made you more anxious before and after the procedure; which hurts more; which works better; which do you prefer; which is easier; how many lines on the eye chart would you be willing to lose to receive fewer treatments; would you be willing to come in every month for treatment; and which treatment allows you to drive from the office after being treated. Patients were also asked if they would choose monthly injections with better vision compared to 4 visits with focal grid laser and slightly less vision.

Primary outcome measures of this study included which treatment was preferred (lasers or injections), how often patients are willing to be treated, and how much vision they are willing to sacrifice to avoid being treated every month.

All lasers done in the past were performed in a standard modified Early Treatment Diabetic Retinopathy Study (ETDRS) fashion [5]. All intravitreal injections were performed in a standard fashion using American Academy of Ophthalmology (AAO) protocols with topical anesthetic and 5% povidone iodine, without lid speculum, or draping the patient.

Statistical analysis was performed using chi square testing to compare choices on survey questions with different demographic subgroups. Baseline patient characteristics were summarized using frequencies and percentages, and patient treatment preference was estimated for each demographic subgroup. Association of demographic features with treatment preference were assessed using chi square tests. *p* values of 0.05 or less (two sided) were considered statistically significant.

## 3. Results

A total of 66 patient statuses post treatment with focal grid laser and bevacizumab injections for diabetic macular edema completed the survey. The mean patient age was 63 (range 30–91), with 34 (52%) males and 32 (48%) females participating. 35 (54%) patients were Caucasian, and 31 (46%) were African American. Patients had been diabetic for a mean of 20.7 years (range 3–53 years) and had been seeing a retina specialist (JOM) for a mean of 4.4 years (range 1, visit 20 years). Of the 66 patients, 45% (30/66) were insured by Medicare or Medicaid, 18% (12/66) were insured by BlueCross, 30% were insured by others, and 6% were uninsured. Patients surveyed had received a mean of 4.82 lasers (range 1–19) and 4.86 injections (range 1–25).

Results showed that 56% (37/66) of patients preferred injections, 33% (22/66) preferred focal grid laser, and 11% (7/66) had no preference. Regarding effectiveness of treatment of either focal grid laser or injections, 38% (25/66) of patients found no difference regarding effectiveness of treatment of either focal grid laser or injections, 36% (24/66) of patients reported that focal grid laser works better, and 25% (17/66) reported that injections work better. Regarding pretreatment anxiety, 56% (37/66) reported that injections caused more pretreatment anxiety. 50% (33/66) of patients also reported that focal grid laser treatment was easier for them to undergo, second to 38% (25/66) who reported injections were easier for them to undergo, and 12% (8/66) who had no preference.

Regarding vision, 83% (55/66) of patients said that they would be willing to have 15-16 injections to gain 2 lines of vision. When asked to pick monthly injections with slightly better vision or 4 visits with laser and slightly less vision, 78% (52/66) chose monthly injections with better vision, as opposed to 21% (14/66) who chose fewer visits with less visual gain. 86% (56/66) of patients reported that they would be willing to sacrifice zero lines on the Snellen eye chart to receive 4 lasers as opposed to 15-16 shots, followed by 8% (5/66) who answered that they would sacrifice 2 lines and 6% (4/66) who would sacrifice one line on the Snellen eye chart. Regarding sacrificing lines on the Snellen eye chart in order to receive fewer treatments, 91% (60/66) said that they would sacrifice zero lines on the eye chart, and 76% (50/66) of patients would be willing to come in 12 times a year to receive treatment in order to maintain their vision.

Regarding employment status, 17/66 (26%) were employed, while 49/66 (74%) were unemployed. Mean BCVA was 20/70 for the employed group and 20/80 for the unemployed group, showing no statistical significance. The BCVA did not correlate with employment status.

Regarding those patients who were willing to sacrifice BCVA to receive fewer treatments, the mean BCVA was 20/60 and they had received a mean of 6.7 laser treatments and 3.5 injections. This was not statistically different from patients unwilling to sacrifice BCVA to receive fewer treatments (mean BCVA 20/70, mean lasers 4.82, mean injections 4.86).

Gender was a statistically significant factor as it pertains to pain tolerance and pain perception for focal grid laser and injections. Out of 18 patients who reported that focal grid laser hurt more than injections, 67% (12/18) of those were male patients. Furthermore, of the 27 patients who reported that either focal grid laser or injection was painful, only 37% (10/27) of those were male and 63% (17/27) were female. Males overwhelmingly chose fewer treatments and poorer vision as opposed to more treatments and better vision in a ratio 11 to 3 compared to female patients. Of those patients preferring fewer treatments, even if that meant having to sacrifice vision, 76% were male (*p* = 0.0225).

The questionnaire surveyed demographic factors, and 17% (10/59) of patients reported having transportation problems. 75% (49/66) of patients reported being unemployed, 45% (30/36) did not attend college, and 40% (27/66) also reported being on disability. There was a statistical significance between being unemployed and posttreatment anxiety for both lasers and injections. Those unemployed had more anxiety with receiving injections (*p* = 0.036). There was also statistical significance between employment and treatment preference between focal grid laser and injection. Those unemployed preferred to have laser treatment over injections compared to those with a job (*p* = 0.0139). Regarding pain after either laser or injections, males were more likely to have pain than females (*p* = 0.05).

## 4. Discussion

Diabetic macular edema is one of the leading causes of vision loss in diabetics in the United States. DME has a vast impact on the quality of life in patients suffering with this disease due to visual acuity loss over time. This vision loss can lead to inability to drive or hold a job, leading to psychological symptoms of depression and anxiety. Rees et al. examined the association between vision loss and DME with psychological outcomes such as symptoms of anxiety and depression in patients with diabetes. In their study, greater depressive symptoms correlated with more severe diabetic retinopathy, lower education level, poorer glucose control, and longer duration of diabetes, and greater symptoms of anxiety were reported with lower education level [3]. Our findings regarding employment and treatment anxiety correlated with these results. 75% (49/66) of patients surveyed reported being unemployed. This extremely high rate is possibly due to poor health secondary to their disease, the number of doctor visits per month, and the location of the study being rural Alabama. There was a statistical significance between being unemployed and posttreatment anxiety, showing that those who are unemployed show more anxiety with injections (*p* = 0.036). There was also statistical significance between employment and treatment preference between focal grid laser and injection. Those unemployed preferred to have laser treatment over injections compared to those with a job (*p* = 0.0139). This strong correlation between unemployment, anxiety, and treatment choice may lead retina physicians to consider these associations prior to treatment when considering lasers or injections for DME.

Our questionnaire surveyed demographic factors, and 17% of patients (10/59) reported having transportation problems. This could be due to socioeconomic status or a result of losing vision and the inability to drive. 75% (49/66) of patients reported being unemployed, 45% (30/36) did not attend college, and 40% (27/66) also reported being on disability. Lower socioeconomic status may be substantial in contributing to transportation problems, lack of education of their disease, lack of a college education, disability, and low overall health management, leading to poorer vision and higher depressive symptoms long term. Our findings corroborate Rees et al. [3]. These results lead us to believe that socioeconomic status, transportation issues, education, employment, and disability are all significant factors seen in our survey that can be linked to severity of DME requiring many years of treatment.

We found that the majority of patients surveyed were unemployed, which may be due to a number of factors including the number of doctor visits they must make per month, diabetes affecting their ability to be employed, and their relatively poor visual acuity requiring frequent treatments by the ophthalmologist. We found no correlation between employment status and patient's BCVA. Therefore, our patient's visual acuity alone is not the determining factor for their employment status.

Those patients willing to sacrifice vision to receive fewer treatments had no difference in BCVA compared to those patients unwilling to sacrifice vision for fewer treatments. We found that there was no difference between number of treatments received by both groups. This implies that visual acuity and number of treatments received have no bearing on those patients who are willing to sacrifice vision to receive fewer treatments. Gender played a role as we will discuss in the following paragraph.

Gender differences were also statistically significant with regard to patient treatment preference. When asked if patients preferred an injection once a month for better vision or 4 laser treatments per year and slightly worse vision, 14/66 (21%) chose 4 lasers and slightly worse vision. Males overwhelmingly chose fewer treatments and poorer vision as opposed to more treatments and better vision. Of those patients preferring fewer treatments, even if that meant having to sacrifice vision, 76% were male (*p* = 0.0225). Regarding pain after either laser or injections, males were also more likely to have pain than females (*p* = 0.05). These findings lead us to believe that females have a higher pain tolerance when it comes to focal grid laser and injection for DME when compared to males. This supports the pain perception study performed by Frot et al. [6]. This study found that pain intensity, unpleasantness, and anxiety, especially as it pertains to the face, are much higher in men than in women [6]. Although men have lower overall pain ratings, they reported more pain-related anxiety and showed a more positive correlation between anxiety and pain intensity and unpleasantness than women. These results also explain why 56% (10/18) of men reported having posttreatment anxiety regarding injections as compared to 44% in women.

Regarding vision, 91% (60/66) said that they would sacrifice zero lines on the eye chart and 76% (50/66) of patients would be willing to come in 12 times a year to receive treatment in order to maintain their vision. Previous studies have identified a link between visual impairment, anxiety, and depressive symptoms [3, 7–9]. Our study lends further credence that sight is indeed precious and patients recognize this fact. Our patients, in general, are unwilling to sacrifice any vision regardless of the number of treatments or the type of treatments they must receive.

In conclusion, this is the first study to evaluate patient preferences for treatment with focal grid laser and intravitreal injection in diabetics with DME. The results of the survey show that there is no statistically significant difference between patient preferences for laser or intravitreal injection treatment, 76% of patients are willing to be treated a maximum amount of times in order to maintain their vision, and 91% would not give up any vision to receive fewer treatments. Patients express a strong overall preference for treatment schemes that allow the highest degree of visual acuity and stability that can be achieved. Treatment schemes that result in optimal visual outcomes are preferred, even if it involves a high treatment burden. A significant correlation was found between unemployment and gender with regard to treatment preference, suggesting that demographics also play a role in patient preferences toward DME treatment.

## Figures and Tables

**Table 1:** Patient DME survey.

Patient Name:	
Age:	
Race:	
Gender:	
Job/No Job:	
Did they attend college:	
How long they've been diabetic:	
How long they have seen Dr. Mason:	
Vision:	
How many doctors do they have:	
How many lasers have they received:	
How many shots have they received:	
Insurance carrier:	
1. Which made you more anxious before you had either procedure (circle one): laser or shot	
2. Which makes you more anxious now that you've had both (circle one): laser or shot	
3. Which works better? (circle one) laser or shot	
4. Which do you prefer? Laser or shot Why?	
5. Which is easier for you? (circle one) laser or shot	
6. If three lasers did not make you see quite as well as 15-16 shots, which would you pick? (circle one) Lasers or shots	
7. How many lines on the eye chart would you be willing to give up in order to receive just 3 lasers as opposed to 15-16 shots?	
8. Would you be willing to lose one line of vision on the eye chart so you didn't have to come in every month for a shot? Y N	
9. Would you be willing to lose two lines of vision on the eye chart so you didn't have to come in every month for a shot? Y N	
10. Would you be willing to lose three lines of vision on the eye chart so you didn't have to come in every month for a shot? Y N	
11. Is it worth 15-16 shots to gain on average 2 lines of vision? Y N	
12. On a scale of 1-5, what is the chance you can visit for 15-16 shots over 2 years? (1=most likely; 5=least likely)	
13. On a scale of 1-5, what is the chance you can visit for 7 shots and 3 lasers over 2 years? (1=most likely; 5=least likely)	
14. On a scale of 1-5, what is the chance you can visit for 4 lasers over 2 years? (1=most likely; 5=least likely)	
